# Deep learning-driven macroscopic AI segmentation model for brain tumor detection via digital pathology: Foundations for terahertz imaging-based AI diagnostics

**DOI:** 10.1016/j.heliyon.2024.e40452

**Published:** 2024-11-15

**Authors:** Myeong Suk Yim, Yun Heung Kim, Hyeon Sang Bark, Seung Jae Oh, Inhee Maeng, Jin-Kyoung Shim, Jong Hee Chang, Seok-Gu Kang, Byeong Cheol Yoo, Jae Gwang Kwon, Jungsup Byun, Woon-Ha Yeo, Seung-Hwan Jung, Han-Cheol Ryu, Se Hoon Kim, Hyun Ju Choi, Young Bin Ji

**Affiliations:** a Advanced Photonics Research Institute (APRI), Gwangju Institute of Science and Technology (GIST), Gwangju, 61005, Republic of Korea; b Gimhae Biomedical Center, Gimhae Biomedical Industry Promotion Agency (GBIA), Gimhae, 05969, Republic of Korea; c DX Business Division, Deepnoid.Inc, Seoul, 08376, Republic of Korea; d YUHS-KRIBB Medical Convergence Research Institute, Yonsei University College of Medicine, Seoul, 03722, Republic of Korea; e Department of Neurosurgery, Brain Tumor Center, Severance Hospital, Yonsei University College of Medicine, Seoul, 03722, Republic of Korea; f Department of Artificial Intelligence Convergence, Sahmyook University, Seoul, 01795, Republic of Korea; g Department of Pathology, Severance Hospital, Yonsei University College of Medicine, Seoul, 03722, Republic of Korea

## Abstract

We used deep learning methods to develop an AI model capable of autonomously delineating cancerous regions in digital pathology images (H&E-stained images). By using a transgenic brain tumor model derived from the TS13-64 brain tumor cell line, we digitized a total of 187 H&E-stained images and annotated the cancerous regions in these images to compile a dataset. A deep learning approach was executed through DEEP:PHI, which abstracts Python coding complexities, thereby simplifying the execution of AI training protocols for users. By employing the Image Crop with Mask technique and patch generation method, we not only maintained an appropriate data class balance but also overcame the challenge of limited computing resources. This approach enabled us to successfully develop an AI training model that autonomously segments cancerous areas. This AI model enables the provision of guiding images for determining cancerous areas with minimal assistance from neuropathologists. In addition, the high-quality, large dataset curated for training using the proposed approach contributes to the development of novel terahertz imaging-based AI cancer diagnosis technologies and accelerates technological advancements.

## 1 Introduction

Cancer remains one of the major public health issues globally, with its incidence and mortality rates persistently rising due to population growth, increasing average lifespan, and lifestyle changes [1]. Consequently, the importance of research in cancer prevention and early diagnosis and the development of effective treatments is attracting increasing attention. Traditional medical imaging technologies, such as magnetic resonance imaging (MRI) and computed tomography (CT), are frequently utilized in cancer diagnosis. However, there are several challenges associated with detecting or diagnosing cancer during surgery. Cutting-edge optical technologies, including fluorescence imaging [2], optical coherence tomography (OCT) imaging [3], Raman spectroscopy imaging [4], and terahertz imaging [[5], [6], [7], [8]], are emerging as cancer diagnostic tools. These technologies, which are compact and easy to use in the operating room, are increasingly being utilized in practical clinical applications [[9], [10], [11]].

In particular, visual distinction is a major challenge in treating malignant brain tumors, making the use of MRI-based neuronavigation devices and 5-aminolevulinic acid (ALA)-induced fluorescent imaging techniques essential. These techniques have improved treatment outcomes of surgery. However, unmet medical needs persist due to issues such as brain shifts in neuronavigation devices and the absence of fluorescence expression in fluorescent imaging techniques for low-grade brain tumors [12]. Currently, the major challenge during surgical treatment is accurately distinguishing low-grade malignant brain tumors. Recent reports suggest that low-grade brain tumors can be identified through advanced medical imaging technology using terahertz electromagnetic waves [7]. Therefore, it is anticipated that this technology can greatly improve cancer treatment outcomes.

However, manual analysis of terahertz and optical imaging data is both time-consuming and susceptible to inconsistencies or errors when conducted by nonexperts. To address these challenges, the deployment of artificial intelligence (AI) technologies is proposed. Recent studies have reported that leveraging AI technology with U-Net CNN-based deep learning has enhanced the accuracy of brain tumor segmentation in MRI images and reduced diagnostic time [13]. AI technologies can efficiently process terahertz and optical images, enabling a trained cancer diagnostic model to provide accurate and consistent diagnoses [14]. Thus, these technologies have the potential to enhance cancer diagnosis during surgery, offering real-time, dependable support.

However, the development of AI-based cancer diagnostic technologies necessitates an ample supply of high-quality training datasets, with a significant emphasis on acquiring datasets that are precisely annotated to delineate cancerous tissues [15]. To develop a specialized AI algorithm for cancer detection through terahertz imaging, it is imperative to have access to digital pathology datasets containing images with accurately labeled cancerous regions, ensuring that these algorithmically inferred regions align with actual histopathological findings. However, generating such annotated digital pathology datasets requires the advanced expertise and experience of medical professionals, rendering the process both time-consuming and labor-intensive [16].

The development of AI-driven cancer diagnostic technologies that leverage terahertz imaging could significantly enhance cancer care by facilitating real-time diagnosis during surgeries for tumors of varying grades, from high-to low-grade brain tumors [7]. However, the acquisition and utilization of digital pathology images with annotated cancerous regions, which are essential for developing accurate and reliable models, are hindered by significant obstacles, such as the scarcity of pathologists, lack of collaboration, and large volume of digital pathology data. To address these challenges, the development of AI-driven automatic cancer region segmentation technologies based on digital pathology is a critical priority. Moreover, most current studies on digital pathology-based cancer diagnosis in deep learning focuses intensively on learning from the morphology of individual cell nuclei and the microscopic changes surrounding them. In contrast, AI cancer diagnostics using terahertz imaging require digital pathology images that are annotated at the macroscopic level to identify broader distributions of cancerous areas. Unfortunately, studies providing such macroscopically annotated digital pathology images for terahertz-based AI-based cancer diagnostics are exceedingly rare.

The objective of this study is to develop an AI-based automatic cancer region segmentation model using digital pathology to obtain high-quality data that is annotated with cancer regions, which is essential for the development of terahertz imaging-based AI-based cancer diagnostic models. To achieve this goal, whole-slide images (WSIs) from transgenic mouse brain tumor models were used. The proposed model is expected to reduce the workload and fatigue of histopathology experts and medical imaging specialists. An additional aim of this study is to leverage state-of-the-art optical technologies, including terahertz imaging, and accelerate the development of advanced terahertz and optical imaging-based AI-based cancer diagnostic technologies by providing researchers with extensive digital pathology images annotated with cancerous regions.

## 2 Materials and methods

In this study, we constructed a TS model using primary tumor cells, TS13-64, derived from a patient with glioblastoma (GBM). TS13-64 was obtained from fresh glioblastoma (GBM) tissue samples, under approval from the Institutional Review Board of Yonsei University College of Medicine (IRB No. 4-2021-1319). For TS culture, the cells were maintained in TS complete media, which consisted of DMEM/F-12 (Mediatech, Manassas, VA), 1x B27 supplement (Invitrogen, San Diego, CA), and 20 ng/mL each of bFGF and EGF (Sigma-Aldrich, St. Louis, MO) [17]. The cell lines used in our study were free of mycoplasma and all experiments were conducted with mycoplasma-free cells.

### 2.1 Transgenic brain tumor models

**Fig. 1:**
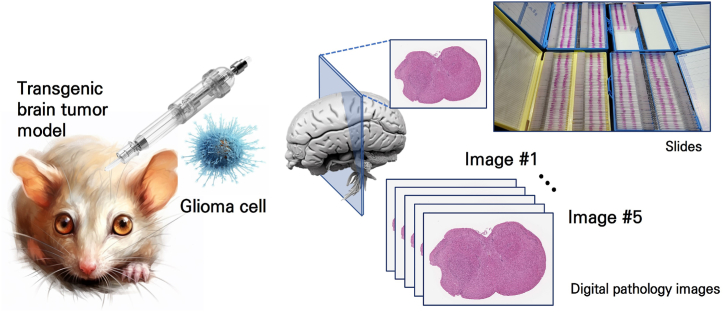
Schematic diagram of the process for generating a transgenic brain tumor animal model in mice and obtaining H&E-stained images for the development of an AI training model for automatic cancer segmentation. Following injection of the TS13-64 cell line and induction of tumor formation, the brains were excised, tissue-stained slides were prepared, and digital pathology images were generated.

To acquire data with characteristics similar to those of human malignant gliomas, we employed xenograft mouse models of brain tumors. Male athymic BALB/c nude mice aged 8 weeks (Orient Bio, Seongnam-Si, Republic of Korea) were used for the glioma transplantation experiments. The mice were housed in microisolator cages under sterile conditions and monitored for a minimum of one week prior to the initiation of the experiments to ensure their health status. Environmental parameters such as temperature, lighting, and humidity were centrally regulated. All the experimental procedures were executed in compliance with the guidelines established by the Institutional Animal Care and Use Committee (IACUC) of Yonsei University College of Medicine, with prior approval. The mice were anesthetized via intraperitoneal injection of a solution containing Zoletil® (30 mg/kg; Virbac Korea, Seoul, South Korea) and xylazine (10 mg/kg; Bayer Korea, Seoul, South Korea). A guide-screw system was used to implant bolts into the skulls of each mouse. For each of the 50 mice, 2x10^5 TS13-64 cells were administered into the right frontal lobe at a depth of 4.5 mm using Hamilton syringes [18,19]. TS13-64 cells were injected into eight mice concurrently using a multiple micro-infusion syringe pump (Harvard Apparatus, Holliston, MA) at a rate of 0.5 μl/min. The body weight of the mice was monitored every other day. Mice were euthanized using CO2 gas according to protocol if their body weight decreased by more than 15 % from their maximum weight. Fig. 1 shows a schematic representation the process for obtaining hematoxylin and eosin (H&E)-stained images for deep learning via a transgenic brain tumor model.

### 2.2 H&E-stained pathology slides

A total of fifty mice were subjected to intracranial inoculation with the TS13-64 cell line to induce brain tumors. After inoculation, the mice were carefully monitored for the emergence and progression of intracerebral tumor growth via MR images. Of the initial fifty mice, 35 developed brain tumors.

For histopathological analysis, the mice with induced brain tumors (n = 35) were euthanized, followed by cautious encephalic extraction. The excised brain tissues were fixed in 10 % neutral-buffered formalin and then embedded in paraffin blocks. Serial sections with a thickness of 5 μm were procured from the paraffin-embedded samples at intervals of 200 μm. Sections with an adequate presence of neoplastic infiltration were selectively included for further analysis, while those devoid of neoplastic cells were excluded. H&E staining was carried out in accordance with standardized protocols to facilitate histomorphological evaluation of the neoplasm and the surrounding cerebral tissue architecture. The H&E-stained slides were subjected to microscopic inspection for histopathological assessment.

### 2.3 High-magnification digital scanners

The H&E-stained tissue slides were digitized for further analysis. For this purpose, we utilized an Aperio CS Digital Scanner from Leica Biosystems (Aperio CS2, Nussloch, Baden-Württemberg, Germany). The scanner was set to a magnification level of 200×, offering a detailed high-resolution histological representation of each slide. The scanned images were reconstructed to produce a large-scale digital histopathological image, each with a file size ranging from approximately 600 MB–900 MB, thereby facilitating an in-depth investigation of the tumor morphology and its surrounding tissue architecture. In total, 187 high-resolution digital histopathological images were acquired through this process. This digital reconstruction process enabled us to observe and analyze the complex histopathological features of brain tumors within the context of the native tissue environment. In addition, the digitization process made it easier to label cancerous areas in the H&E-stained tissue images, thereby simplifying the acquisition of the dataset for deep learning.

### 2.4 Generating labeled XML data with brain tumor regions (generating a dataset for the AI model)

The acquired high-resolution digital histopathological images were used to create a dataset for developing AI-based automated segmentation technology for tumor regions. In collaboration with the Department of Pathology at Yonsei University, a specialized annotation software named Deep:pathology was employed to delineate and label the cancerous regions within the images. An experienced pathologist performed the initial annotations. The cancerous regions were meticulously demarcated and annotated to generate preliminary ground truth labels. The H&E-stained images were provided to the pathologists with file names consisting of identical prefixes and numbered combinations, with the cell line type and tumor inclusion status fully blinded. After the initial labeling, random patch extraction was applied to balance the ‘background,’ ‘normal,’ and ‘tumor’ regions, taking care to prevent biased learning outcomes. Both the experienced pathologist and the research participated in a collaborative review process that entails thorough cross-checking and consultation to examine and refine the annotations.

After collaborative expert consensus, the refined annotations were stored in XML format, capturing the spatial characteristics of the tumor regions with high precision. The high-resolution digital histopathological images and carefully curated XML files were included in a comprehensive dataset that was utilized for training the deep learning algorithm.

### 2.5 AI development solution for deep learning (DEEP:PHI & Python)

We used a meticulously curated dataset derived from high-resolution digital histopathological images and precisely annotated XML files to develop an automated cancer region segmentation model through a deep learning approach. For this study, we employed DEEP:PHI (Seoul, Republic of Korea), a cutting-edge AI development solution created by Deepnoid. DEEP:PHI features a graphical user interface (GUI) that abstracts Python-based coding, making it easier for users to execute AI training protocols. We imported the original dataset into the DEEP:PHI environment and initiated a custom training schema for neoplastic region segmentation. In terms of a neural network architecture, we chose the U-Net convolutional neural network (CNN) [20], which is known for its proficiency in biomedical image segmentation. The effectiveness of U-Net is attributed to its symmetrical and expansive topology, adeptly capturing both the macroscopic contextual and microscopic granular characteristics in images. We partitioned our dataset into training, validation, and testing sets at a ratio of 8:1:1, ensuring robust evaluation and unbiased performance metrics. The learning process was closely monitored to ensure the model's effectiveness, and the performance of the model was carefully evaluated via two metrics. The first metric, the intersection over union (IoU), quantitatively evaluates the accuracy of the segmented areas by comparing the overlap between the ground truth and the predicted segments. The second metric, the Dice score, measures the similarity between the predicted segmentation result and the ground truth, effectively emphasizing the accuracy of the true positive predictions.

## 3 Results

### 3.1 Results of data training using the image crop with mask (IMCM) function

**Fig. 2:**
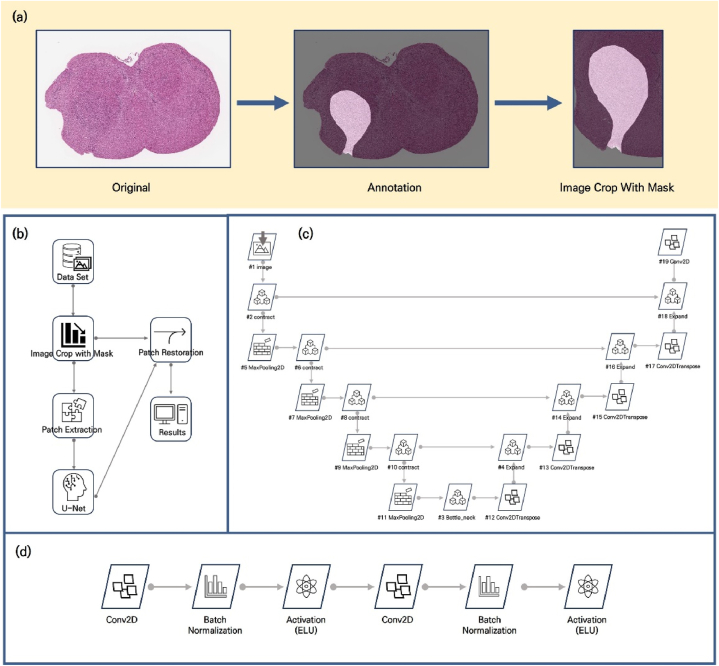
Training process and flow diagram using the image crop with mask (ICWM) function. (a) ICWM generation process. This methodological adaptation allowed us to reduce the required training dataset size. (b) Training flow using DEEP:PHI. (c) Hierarchical structure of the U-Net neural network. U-Net utilizes an encoder-decoder architecture, performing efficient and accurate image segmentation through downsampling and upsampling processes. (d) Flow diagram of each contract module.

By leveraging a dataset comprising 187 paired instances, we endeavored to train an AI model for cancer segmentation via DEEP:PHI. However, computational limitations, worsened by the large size of digital pathology WSI datasets, prevent the full use of WSIs in the training process. To address this limitation, we deployed the image crop with mask (ICWM) function, which is integrated within the DEEP:PHI framework, allowing us to perform targeted cropping of WSIs for training purposes, as depicted in Fig. 2(a). This methodological adaptation allowed us to reduce the required training dataset size to approximately one-seventh of its original size. Furthermore, this process enables a more precise adjustment of the ratio between cancerous regions and healthy tissue than can be achieved with direct WSI application, optimizing class balance for AI model training.

Despite the use of the ICWM function to address computational limitations, training was ultimately carried out with only 53 datasets due to ongoing computational constraints. The training flow, depicted in Fig. 2 (b), involves segmenting each dataset into 512 × 512 pixel patches after ICWM processing, training via the well-known U-Net convolutional neural network architecture, and then reassembling the patches to conclude the training process. The patch size was set to 512 pixels with a stride of 256, which defines the step size between patches, to increase the training efficiency. The model consists of 4 stages, with 64, 128, 256, and 512 filters used in each stage. In the bottleneck section, 1024 filters are used. Training was conducted over a total of 50 epochs with a batch size of 16, learning rate of 0.001, utilizing Adam optimizer to achieve reliable results and allow for early termination of the training upon obtaining satisfactory outcomes [21]. The hierarchical structure of the U-Net used in Fig. 2 (C) is diagrammatically represented to explain its configuration. Each contract module consists of two convolutional 2D layers, two batch normalization layers and two activation layers as shown in Fig. 2 (D). Each convolutional 2D layers has same number of filters as that of stages. And we used ELU (exponential linear unit) function [22] for each activation layer. Each function is defined as:

(1) $$\begin{array}{c} f_{ELU}=\left\{ \begin{array}{c} x,\\ e^{x}-1, \end{array}\right.\;\begin{array}{c} x\geq 0\\ x<0 \end{array} \end{array}$$

(2) $$\begin{array}{c} f_{ReLU}=\left\{ \begin{array}{c} x,\\ 0, \end{array}\right.\;\begin{array}{c} x\geq 0\\ x<0 \end{array} \end{array}$$

Bottle-neck module and Expand module have same flow as contract module, except activation layers use ReLU (rectified linear unit) function.

**Fig. 3:**
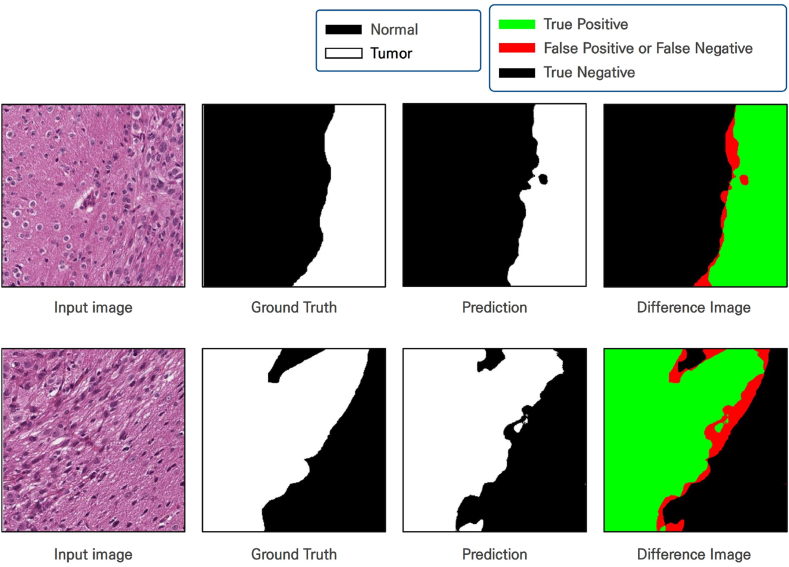
Outcomes of the patch-based training are presented as images. The ground truth image indicates the cancer regions in the input image, while the prediction represents the cancer regions identified by the trained model. The difference image visualizes the discrepancies between the ground truth cancer regions and the predicted regions. It is confirmed that the trained AI model can accurately predict the cancer regions in comparison to the ground truth.

Table 1 presents a summary of the training data results and validation outcomes in a chart, with high Dice scores of 0.9751 and 0.9631 for the normal and tumor categories in the validation dataset, respectively. In the context of AI training with medical data, the performance of the trained model was evaluated on the basis of Dice score, which emphasizes the importance of true positive predictions by assigning appropriate weights. Fig. 3 shows the outcomes of the patch-based training as images. Compared with the ground truth images, the trained model clearly predicts the outcomes well.

**Table 1:** Evaluation of the performance of the proposed model (ICWM).

Process	Class	Dice $\left(\frac{2TP}{2TP+FP+FN}\right)$	IoU $\left(\frac{{TP\;}^{a}}{TP+{FP}^{\;b}+{FN\;}^{c}}\right)$
Training	Normal	0.9994	0.9989
	Tumor	0.9992	0.9983
Validation	Normal	0.9751	0.9497
	Tumor	0.9631	0.9241

^a^True positive.

^b^False positive.

^c^False negative.

### 3.2 Results of data training with patch generation preprocessing

Promising training outcomes are achieved by implementing the ICWM technique. However, the full utilization of the 187 paired datasets was hindered by computational resource limitations. Additionally, when deploying the trained model in practical applications, there is a potential issue in that to fully leverage the training performance, the provided data must be cropped to maintain class balance akin to that in the trained dataset. This limitation necessitated the exploration of an alternative method for data preprocessing prior to training.

**Fig. 4:**
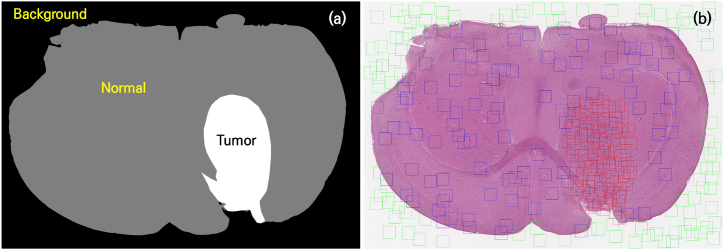
Patch generation process for the random creation of patches to mitigate computational resource constraints and achieve appropriate class balancing. (a) Schematic diagram categorized into three classes: background, normal and tumor. (b) The positions of randomly generated patches of background (green rectangle), normal (blue rectangle) and tumor (red rectangle). The criteria for classifying individual patches were based on whether the labeled training area constituted more than 60 % of the total pixels (512 × 512) in each patch, at which point the patch was classified into the respective class. To avoid extracting the same area multiple times, we excluded patches if the Euclidean distance between each existing patch was less than 256.

Given that different cancerous tissues in the same brain tumor specimen can exhibit similar morphological features, it is crucial to avoid data bias and enhance adaptability for a diverse range of new data by utilizing datasets from all available cases. The ICWM method was limited to using only 53 datasets because of computational resource constraints. To fully leverage all 187 datasets and address the issue of class imbalance in a computing resource-constrained environment, data preprocessing was performed via Python programming by generating patches categorized into three classes—background, normal, and tumor—on the basis of the labeled dataset shown in Fig. 4 (a). First, we converted the image to grayscale and obtained binarized image by applying Otsu's thresholding that calculates the optimal threshold for maximizing the separability between the background and the tissues, including tumor [23]. Second, the remaining area, excluding the background and tumor-labeled regions, is designated as the normal region. The criteria for classifying individual patches were based on whether the labeled training area constituted more than 60 % of the total pixels (512 × 512) in each patch, at which point the patch was classified into the respective class. For each class, 100 patches were randomly selected by using NumPy's random module to construct a dataset, and a total of 49,915 patches (18,700 background, 18,700 normal, and 12,515 tumor patches) were obtained for the training process. To avoid extracting the same area multiple times, we excluded patches if the Euclidean distance between each existing patch was less than 256. The results of the randomly extracted patches are presented in Fig. 4 (b).

**Fig. 5:**
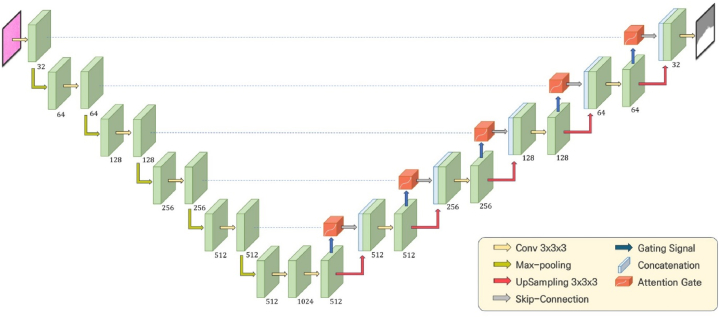
Network architecture diagram for the proposed model (Attention U-Net). Attention U-Net is a model an architecture based on the U-Net model with added attention mechanisms. This model learns to suppress relatively less important areas in the input images while enhancing regions with important features. The model consists of 5 stages, with 32, 64, 128, 256, and 512 filters used in each stage. In the bottleneck section, 1024 filters are used. Additionally, to minimize overfitting, dropout layers are added after each max pooling operation, and batch normalization layers are added after each convolution operation.

**Fig. 6:**
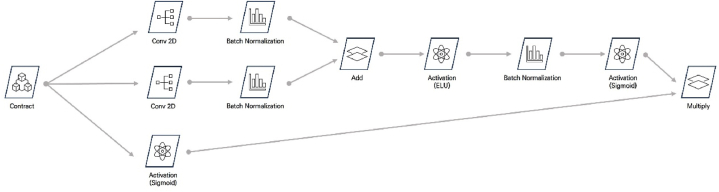
Flow diagram of attention module applied in our Attention U-Net.

Finally, the values of each patch were rescaled to 0–1. We chose a DEEP:PHI built-in model: Attention U-Net (see Fig. 5). Attention U-Net is a model an architecture based on the U-Net model with added attention mechanisms (see Fig. 6). This model learns to suppress relatively less important areas in the input images while enhancing regions with important features [24]. The model consists of 5 stages, with 32, 64, 128, 256, and 512 filters used in each stage. In the bottleneck section, 1024 filters are used. Each convolutional 2D layer has same number of filters as that of stage. Additionally, to minimize overfitting, dropout layers are added after each max pooling operation, and batch normalization layers are added after each convolution operation During training, the hyperparameters were set as follows: 80 epochs, batch size of 16, Dice loss as the loss function, Adam optimizer with an initial learning rate of 0.0005 and linear decay of 0.91.

**Fig. 7:**
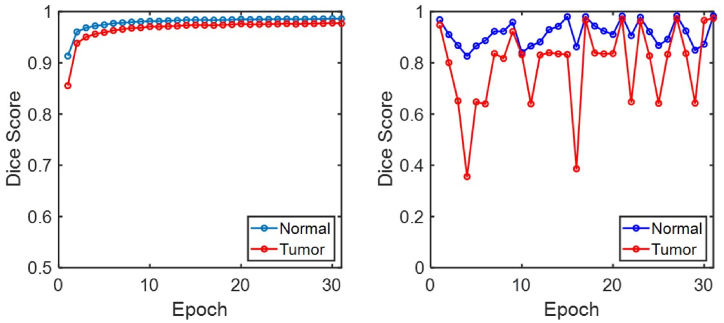
Results of deep learning with the attention U-Net model. (a) Training Dice score; (b) validation Dice score. The training outcomes demonstrate that a high-performance model was developed, achieving a Dice score of 0.9736 with the validation data and 0.9753 with the test data.

Training was stopped at epoch 31 due to the early stopping callback, which monitored validation loss. On average, each epoch was completed in approximately 37 min, resulting in a total training time of approximately 19 h. The training outcomes demonstrate that a high-performance model was developed, achieving a Dice score of 0.9736 with the validation data and 0.9753 with the test data, as shown in Fig. 7 and Table 2.

**Table 2:** Evaluation of the performance of the proposed model.

Process	Class	Dice $\left(\frac{2TP}{2TP+FP+FN}\right)$
Training	Normal	0.9858
	Tumor	0.9768
Validation	Normal	0.9832
	Tumor	0.9736
Test	Normal	0.9851
	Tumor	0.9753

**Fig. 8:**
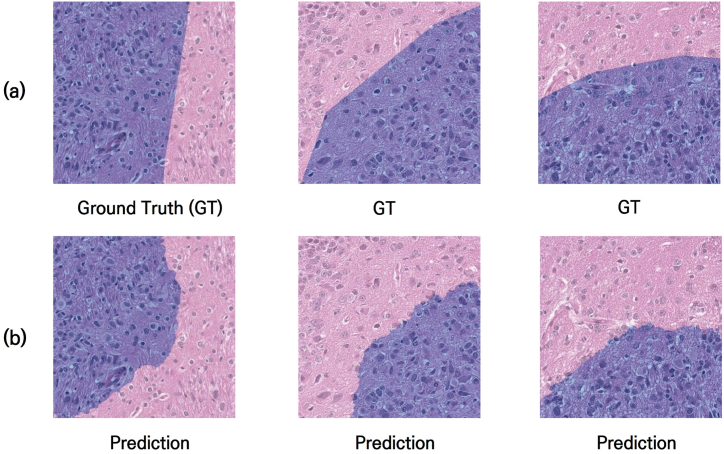
By using a patch generation method, the AI model was trained to effectively predict cancerous regions in images. (a) Ground truth image with labeled cancer regions. The cancer areas are indicated in light purple in the H&E-stained images. (b) Image with cancer regions predicted by the trained AI model. Although the trained model can represent the geometric features of the cancer more precisely, it does not necessarily mean that the AI model can label the cancer areas more accurately, as the actual cancer regions require consideration of various factors, including the shape of cancer cell nuclei, contrast with normal tissues, etc.

Fig. 8 shows images where the trained AI model predicted the cancerous regions well, i.e., outcomes closely resembling the ground truth. The ground truth in Fig. 8(a) was chosen to intentionally highlight areas where detailed labeling was lacking due to human factors while showing that the trained model can depict geometric characteristics more concretely and in detail. However, actual pathological assessments of cancerous regions by medical experts involve not only geometric shapes but also a comprehensive evaluation of contrast with normal regions, the morphology of each cell nucleus, and various other factors, indicating that the actual cancerous areas may be more extensive than those predicted by this AI training model. While further training of a different nature may be required for accurate segmentation down to the level of individual cells, the primary goal of this study was to provide macroscopic cancerous area segmentation images for AI training on the basis of terahertz and optical imaging. Thus, a model capable of providing the necessary level of data for the authors' needs has been successfully developed.

**Fig. 9:**
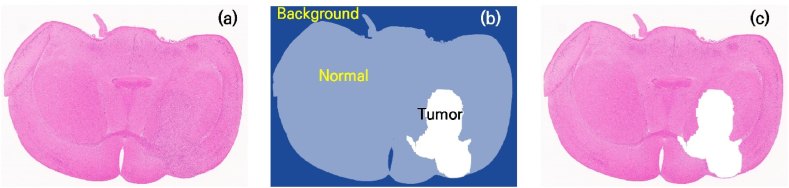
Results from the trained AI model. The cancerous regions in new, untrained H&E-stained images were effectively predicted. (a) Original image, (b) image with background, normal and tumor areas labeled by the trained AI model, and (c) merged image of (a) and the tumor region in (b).

Fig. 9 shows the results of cancer area segmentation via the trained AI model. When the original H&E-stained images were input, the cancer areas were labeled as shown in Fig. 9(b), confirming their well-matched macroscopic alignment with the actual cancer areas, as shown in Fig. 9(c).

## 4 Discussion

The development of deep learning-based terahertz imaging AI for cancer diagnosis could significantly contribute to improving the prognosis of brain tumor patients by enabling the clear differentiation of not only high-grade brain tumors but also low-grade tumors in real time during surgery [7]. However, to achieve the development of a highly effective terahertz imaging-based AI cancer diagnostic technology, it is essential first to establish digital pathology images (H&E-stained images) that can precisely identify cancerous regions within terahertz imaging data. This paper focuses on developing an automated cancer region segmentation technology based on digital pathology, which is a critical component for realizing the authors’ ultimate goal of creating a terahertz imaging-based AI diagnostic model. However, as mentioned in the introduction, there are challenges in acquiring a large number of annotated digital pathology images necessary for AI model development. The primary reason for this difficulty is the shortage of pathology specialists and lack of collaboration.

To address this issue, this study developed an AI model based on deep learning that can automatically annotate cancerous regions in H&E images. Utilizing a dataset consisting of 187 pairs of H&E images and corresponding annotations, we developed an AI model that achieved an excellent performance, with a DICE score of 0.9753. It was also confirmed that the trained AI model accurately annotated cancerous regions in H&E images that were not used during training. Recent research has shown that AI models trained on brain tumor MR images enhance segmentation accuracy and significantly reduce diagnostic time, thereby supporting radiologists [13]. Similarly, this technology is expected to improve brain tumor region segmentation accuracy in H&E images, reduce the potential for human error among neuropathologists, and substantially decrease time consumption.

Nonetheless, one potential limitation of the models is the generalization issue, where performance may degrade when data is input under conditions different from the original data acquisition process. For instance, when obtaining pathology images, differences in microscope or scanner equipment can result in variations in resolution or color. Even with the same equipment, differences in staining techniques or slide preparation processes across institutions can lead to significant variations in data characteristics, potentially impacting model performance. To achieve robust performance across diverse domains, it is essential to collect data from multiple institutions and utilize it for fine-tuning the model. In addition, beyond common data augmentation techniques such as flipping and cropping, applying methods that include intensity variations can further improve the model's generalization performance.

Most previous studies on digital pathology-based cancer diagnosis have focused on learning the morphology of individual cell nuclei and their surrounding microscopic changes [25,26]. However, this study concentrated on automatically annotating macroscopic cancerous regions. The ultimate goal of this technology is to mark cancerous regions in real time during surgery using terahertz imaging, as terahertz imaging is well-suited for visualizing macroscopic tumor regions.

**Fig. 10:**
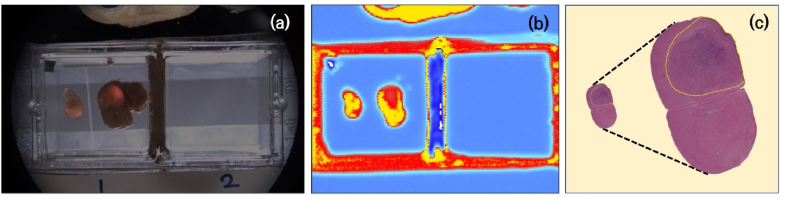
Example of a dataset for AI-based cancer diagnosis training based on terahertz imaging. (a) Merged image of visible light and PPIX fluorescence images. (b) Terahertz image. (c) Digital pathology image with labeled cancerous areas. By training an AI model to identify cancerous areas in terahertz images that most closely match the cancerous regions in (a) and (c), it is possible to develop AI-based cancer diagnosis technology using terahertz imaging.

Nevertheless, there are challenges due to morphological discrepancies between H&E images and actual visible or terahertz imaging, caused by processes such as fixation and slicing during the preparation of H&E slides. Therefore, to further advance terahertz imaging-based AI cancer diagnosis technology, it is crucial to establish optimal threshold values in terahertz imaging that most accurately correspond to cancerous regions, with reference to H&E images (Fig. 10). The proposed AI model can identify cancerous regions with minimal assistance from neuropathologists. As shown in Fig. 10, by providing a high-quality, large-scale dataset for training, this approach is expected to contribute to the development of novel terahertz imaging-based cancer diagnosis technologies and accelerate advancements in this field.

### 4.1 Ethics statement

Approval for specimen collection and analysis was obtained from the Institutional Review Board of our institution (4-2021-1319). This study was carried out in accordance with relevant guidelines and ethical regulations of the institutional and national research committee. The participants provided written informed consent, and this study complies with the Declaration of Helsinki.

The care and handling of animals were performed in compliance with relevant international, national, and institutional regulations. The ethical standards of the institution where the research was carried out were adhered to for all animal procedures. The in vivo experiments and animal care procedures received approval from the Committee for the Care and Use of Laboratory Animals at Yonsei University College of Medicine (approval number 2023-0237) and were conducted in accordance with the US National Institutes of Health guidelines for the Care and Use of Laboratory Animals. We adhered to the ARRIVE guidelines for animal reporting.

## CRediT authorship contribution statement

**Myeong Suk Yim:** Writing – original draft, Software, Investigation. **Yun Heung Kim:** Writing – original draft, Methodology, Investigation. **Hyeon Sang Bark:** Writing – original draft, Methodology, Investigation. **Seung Jae Oh:** Methodology, Investigation. **Inhee Maeng:** Methodology, Investigation. **Jin-Kyoung Shim:** Methodology, Investigation. **Jong Hee Chang:** Methodology, Investigation. **Seok-Gu Kang:** Methodology, Investigation. **Byeong Cheol Yoo:** Methodology, Investigation. **Jae Gwang Kwon:** Methodology, Investigation. **Jungsup Byun:** Methodology, Investigation. **Woon-Ha Yeo:** Software, Methodology. **Seung-Hwan Jung:** Software, Methodology. **Han-Cheol Ryu:** Software, Methodology. **Se Hoon Kim:** Writing – review & editing, Methodology, Data curation. **Hyun Ju Choi:** Writing – review & editing, Methodology, Conceptualization. **Young Bin Ji:** Writing – review & editing, Supervision, Project administration, Methodology, Conceptualization.

## Declaration of competing interest

The authors declare that they have no known competing financial interests or personal relationships that could have appeared to influence the work reported in this paper.

## References

[1] Bray F., Ferlay J., Soerjomataram I., Siegel R.L., Torre L.A., Jemal A. (2018). Global cancer statistics 2018: GLOBOCAN estimates of incidence and mortality worldwide for 36 cancers in 185 countries. CA Cancer J. Clin..

[2] Ntziachristos V., Ripoll J., Wang L.V., Weissleder R. (2005). Looking and listening to light: the evolution of whole-body photonic imaging. Nat. Biotechnol..

[3] Huang D., Swanson E.A., Lin C.P., Schuman J.S., Stinson W.G., Chang W., Hee M.R., Flotte T., Gregory K., Puliafito C.A., Fujimoto J.G. (1991). Optical coherence tomography. Science.

[4] Kong K., Kendall C., Stone N., Notingher I. (2015). Raman spectroscopy for medical diagnostics—from in-vitro biofluid assays to in-vivo cancer detection. Adv. Drug Deliv. Rev..

[5] Woodward R.M., Wallace V.P., Pye R.J., Cole B.E., Arnone D.D., Linfield E.H., Pepper M. (2002). Terahertz pulse imaging of ex vivo basal cell carcinoma. J. Invest. Dermatol..

[6] Ji Y.B., Park C.H., Kim H., Kim S.-H., Lee G.M., Noh S.K., Jeon T.-I., Son J.-H., Huh Y.-M., Haam S., Oh S.J., Lee S.K., Suh J.-S. (2015). Feasibility of terahertz reflectometry for discrimination of human early gastric cancers. Biomed. Opt Express.

[7] Ji Y.B., Oh S.J., Kang S.-G., Heo J., Kim S.-H., Choi Y., Song S., Son H.Y., Kim S.H., Lee J.H., Haam S.J., Huh Y.M., Chang J.H., Joo C., Suh J.-S. (2016). Terahertz reflectometry imaging for low and high grade gliomas. Sci. Rep..

[8] Ji Y.B., Kim J.M., Lee Y.H., Choi Y., Kim D.H., Huh Y.M., Oh S.J., Koh Y.W., Suh J.S. (2019). Investigation of keratinizing squamous cell carcinoma of the tongue using terahertz reflection imaging. J. Infrared, Millim. Terahertz Waves.

[9] Ji Y.B., Lee E.S., Kim S.-H., Son J.-H., Jeon T.-I. (2009). A miniaturized fiber-coupled terahertz endoscope system. Opt Express.

[10] Chen B., Wang H., Ge P., Zhao J., Li W., Gu H., Wang G., Luo Y. (2012). Gross total resection of glioma with the intraoperative fluorescence-guidance of fluorescein sodium. Int. J. Med. Sci..

[11] Stummer W. (2006). Fluorescence-guided surgery with 5-aminolevulinic acid for resection of malignant glioma: a randomised controlled multicentre phase III trial. Lancet Oncol..

[12] Madsen S.J. (2013).

[13] Jwaid W.M., Al-Husseini Z.S.M., Sabry A.H. (2021). Development of brain tumor segmentation of magnetic resonance imaging (MRI) using U-Net deep learning. E. Eur. J. Enterprise Technol..

[14] Hollon T.C., Pandian B., Adapa A.R., Urias E., Save A.V., Shah M.N., Lee R.S. (2020). Near real-time intraoperative brain tumor diagnosis using stimulated Raman histology and deep neural networks. Nat. Med..

[15] Litjens G., Kooi T., Bejnordi B.E., Setio A.A.A., Ciompi F., Ghafoorian M., van der Laak J.A.W.M., van Ginneken B., Sánchez C.I. (2017). A survey on deep learning in medical image analysis. Med. Image Anal..

[16] Janowczyk A., Madabhushi A. (2016). Deep learning for digital pathology image analysis: a comprehensive tutorial with selected use cases. J. Pathol. Inform..

[17] Kong B.H., Park N.R., Shim J.K., Kim B.K., Shin H.J., Lee J.H., Huh Y.M., Lee S.J., Kim S.H., Kim E.H., Park E.K., Chang J.H., Kim D.S., Kim S.H., Hong Y.K., Kang S.G., Lang F.F. (2013). Isolation of glioma cancer stem cells in relation to histological grades in glioma specimens. Childs Nerv Syst.

[18] Kong B.H., Park N.-R., Shim J.-K., Kim B.-K., Shin H.-J., Lee J.-H., Huh Y.-M., Lee S.-J., Kim S.-H., Kim E.-H., Park E.-K., Chang J.H., Kim D.-S., Kim S.H., Hong Y.-K., Kang S.-G., Lang F.F. (2013). Isolation of glioma cancer stem cells in relation to histological grades in glioma specimens. Child's Nerv. Syst..

[19] Kwak J., Shim J.-K., Kim D.S., Lee J.-H., Choi J., Park J., Shin K.-J., Kim S.-H., Kim P., Huh Y.-M., Kim E.H., Chang J.H., Kim S.H., Kang S.-G. (2016). Isolation and characterization of tumorspheres from a recurrent pineoblastoma patient: feasibility of a patient-derived xenograft. Int. J. Oncol..

[20] Ronneberger O., Fischer P., Brox T. (2015). Medical Image Computing and Computer-Assisted Intervention–MICCAI 2015: 18th International Conference, Munich, Germany, October 5-9, 2015, Proceedings, Part III.

[21] Kingma D.P., Ba J. (2014). Adam: a method for stochastic optimization. arXiv preprint arXiv:1412.6980.

[22] Clevert D.-A. (2015). Fast and accurate deep network learning by exponential linear units (ELUs). arXiv preprint.

[23] Otsu N. (1975). A threshold selection method from gray-level histograms. Automatica.

[24] Oktay O. (2018).

[25] Janowczyk A., Madabhushi A. (2016). Deep learning for digital pathology image analysis: a comprehensive tutorial with selected use cases. J. Pathol. Inf..

[26] Litjens G., Sánchez C., Timofeeva N. (2016). Deep learning as a tool for increased accuracy and efficiency of histopathological diagnosis. Sci. Rep..

